# Static Mechanical Stress Induces Apoptosis in Rat Endplate Chondrocytes through MAPK and Mitochondria-Dependent Caspase Activation Signaling Pathways

**DOI:** 10.1371/journal.pone.0069403

**Published:** 2013-07-19

**Authors:** Dechao Kong, Tiansheng Zheng, Ming Zhang, Daode Wang, Shihao Du, Xiang Li, Jiahu Fang, Xiaojian Cao

**Affiliations:** Department of Orthopedics, The First Affiliated Hospital of Nanjing Medical University, Nanjing, China; Instituto Gulbenkian de Ciência, Portugal

## Abstract

Mechanical stress has detrimental effects on cartilaginous endplate chondrocytes due to apoptosis in vivo and in vitro. In this study, we investigated the possible apoptosis signaling pathways induced by mechanical stress in cultured rat cervical endplate chondrocytes. Static mechanical load significantly reduced cell viability in a time- and load-dependent manner, as demonstrated by the Cell Counting Kit-8 (CCK-8) assay. Chondrocyte apoptosis induced by mechanical stress was confirmed by annexin V/propidium iodide (PI) staining and terminal deoxynucleotidyl transferase dUTP nick-end labeling (TUNEL). Western blot analysis revealed that static load-induced chondrocyte apoptosis was accompanied by increased phosphorylation of c-Jun N-terminal kinase (JNK), extracellular signal-regulated kinase 1/2 (ERK1/2), and p38 mitogen-activated protein kinase (MAPK). The loss of mitochondrial membrane potential (ΔΨm), increased Cytochrome *c* release, and activated Caspase-9 and Caspase-3, indicating that the mitochondrial pathway is involved in mechanical stress-induced chondrocyte apoptosis. Treatment with inhibitors of JNK (SP600125), p38 MAPK (SB203580), and ERK (PD98059) prior to mechanical stimulation reversed both the static load-induced chondrocyte apoptosis and the activation of JNK, p38 MAPK, and ERK. Taken together, the data presented in this study demonstrate that mechanical stress induces apoptosis in rat cervical endplate chondrocytes through the MAPK-mediated mitochondrial apoptotic pathway.

## Introduction

Apoptosis of endplate chondrocytes plays an important role in the pathogenesis of intervertebral disc degeneration [1], [2]. Chondrocyte apoptosis can be induced by various stimuli, such as mechanical stress, cytokines, and inflammatory mediators [3], [4], [5], [6], [7], [8]. Endplate chondrocytes are constantly exposed to mechanical loading; therefore, mechanical load is considered to play an important role in the regulation of chondrocyte functions. Previous studies have demonstrated that cyclical loading can stimulate synthesis of the cartilage matrix [9]. In contrast, static loading is associated with cartilage degeneration and chondrocyte apoptosis [4], [10], [11].

Numerous studies have been conducted on the chondrocyte apoptosis induced by various stimuli. However, the signaling cascade of mechanical stress-induced chondrocyte apoptosis remains unclear. Mitogen activated protein (MAP) kinases consist of three subfamilies: extracellular signal-regulated kinase p44/42 MAPK (ERK1/2), p38 MAPK (p38), and c-Jun N-terminal kinase (JNK). These kinases play a key role in regulating a variety of cellular activities, such as cell growth, differentiation, and apoptosis [12], [13], [14]. It has been shown that static compression can stimulate the phosphorylation of ERK1/2, p38 MAPK, and JNK in ex vivo cartilage explants [15]. Activation of ERK1/2, p38 MAPK, and JNK has been reported to participate in chondrocyte apoptosis induced by various stimuli [16], [17].

Apoptosis, or programmed cell death, plays an important role in maintaining homeostasis of normal tissues [18], [19]. Mitochondria are the central regulators of apoptosis and perform this function via disruption of the mitochondrial membrane potential (ΔΨm) and the release of Cytochrome *c*, both of which are under the regulation of the evolutionarily conserved B-cell lymphoma-2 (BCL-2) family proteins [20], [21]. The release of Cytochrome *c* leads to the formation of the Apaf-1/caspase-9 complex, which subsequently leads to the activation of Caspase-9, thereby activating the effector caspases, such as Caspases-3, 6, and 7, that execute the final stages of apoptosis [22].

Investigating mechanical stress-induced endplate chondrocyte apoptosis is crucial to the clinical treatment of spinal degenerative diseases. However, systematic research of the relationship between MAPK pathway activation and mitochondrial control of apoptosis in chondrocytes treated with a static compressive load has not been reported. Therefore, the aim of the present study was to explore the mechanism of static compression induced endplate chondrocytes apoptosis. We examined the effect of 0.5 MPa static loading of endplate chondrocytes for 24 h on the following activities: (1) apoptosis in endplate chondrocytes, (2) the mitochondrial dysfunction and the activation of caspases, and (3) the involvement of MAPK in the static compression-induced apoptosis pathway.

## Results

### Static Compression Decreased Cell Viability in Endplate Chondrocytes

**Figure 1:**
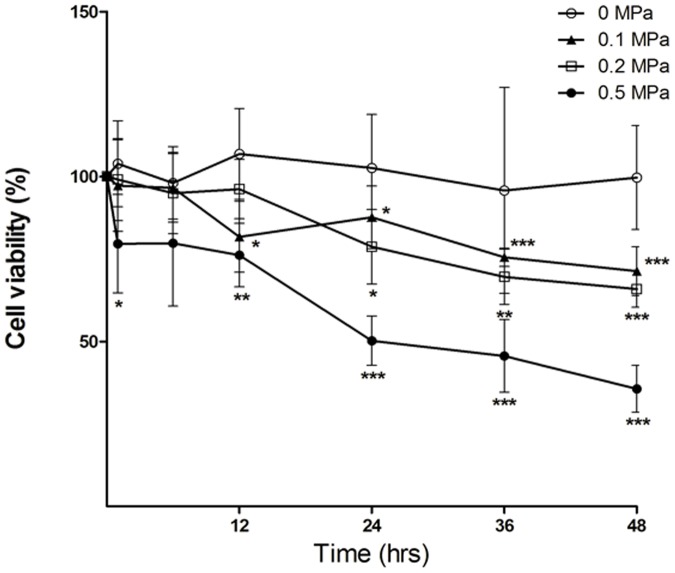
Static mechanical load-induced cell death in endplate chondrocytes. Chondrocytes were exposed to various static loads for 1, 6, 12, 24, 36, and 48 h. Cell viability was detected by using the CCK-8 assay. Each value represents the mean ± SD, n = 3. *p<0.05; **p<0.01; ***p<0.001 compared with the control group.

To investigate the effect of static compression on cell viability, endplate chondrocytes were exposed to various static mechanical loads (0, 0.1, 0.2, and 0.5 MPa) for 1, 6, 12, 24, 36, and 48 h. The results indicated that static loading decreased cell viability in a load- and time-dependent manner (Fig. 1). An effective static compression load of 0.5 MPa for 24 h was chosen for subsequent experiments.

### Static Compression Induces Apoptosis in Endplate Chondrocytes

**Figure 2:**
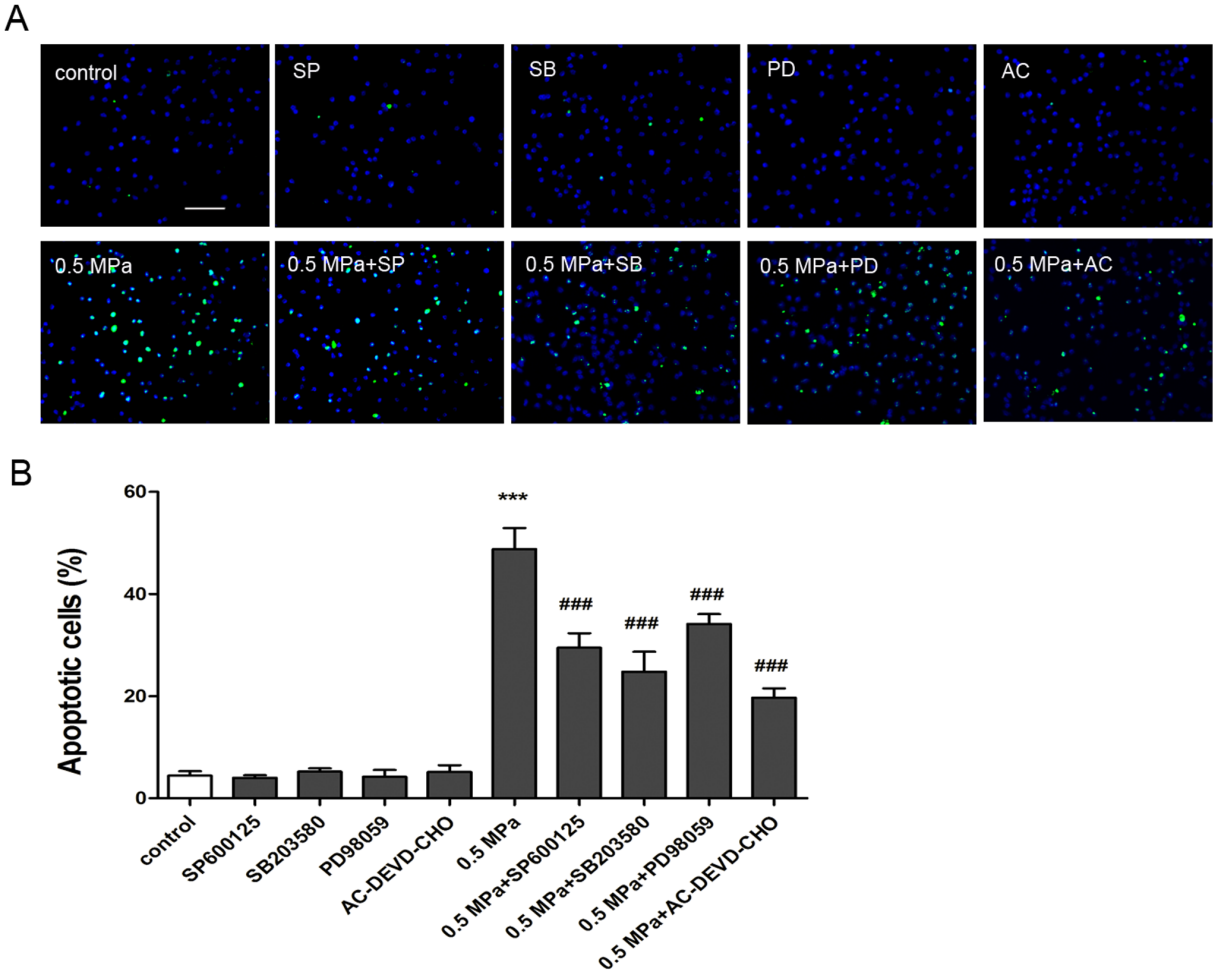
Static compression-induced chondrocyte apoptosis as assessed by TUNEL staining. (A) TUNEL-positive cells (green fluorescence) were observed under a fluorescence microscope. (B) The frequency of apoptotic cells was expressed as a percentage of the total cells. Data are presented as the mean ± SD from three independent experiments. ***p<0.001 versus unloaded cells; ^###^p<0.001 versus loaded cells. Scale bar = 50 µm.

**Figure 3:**
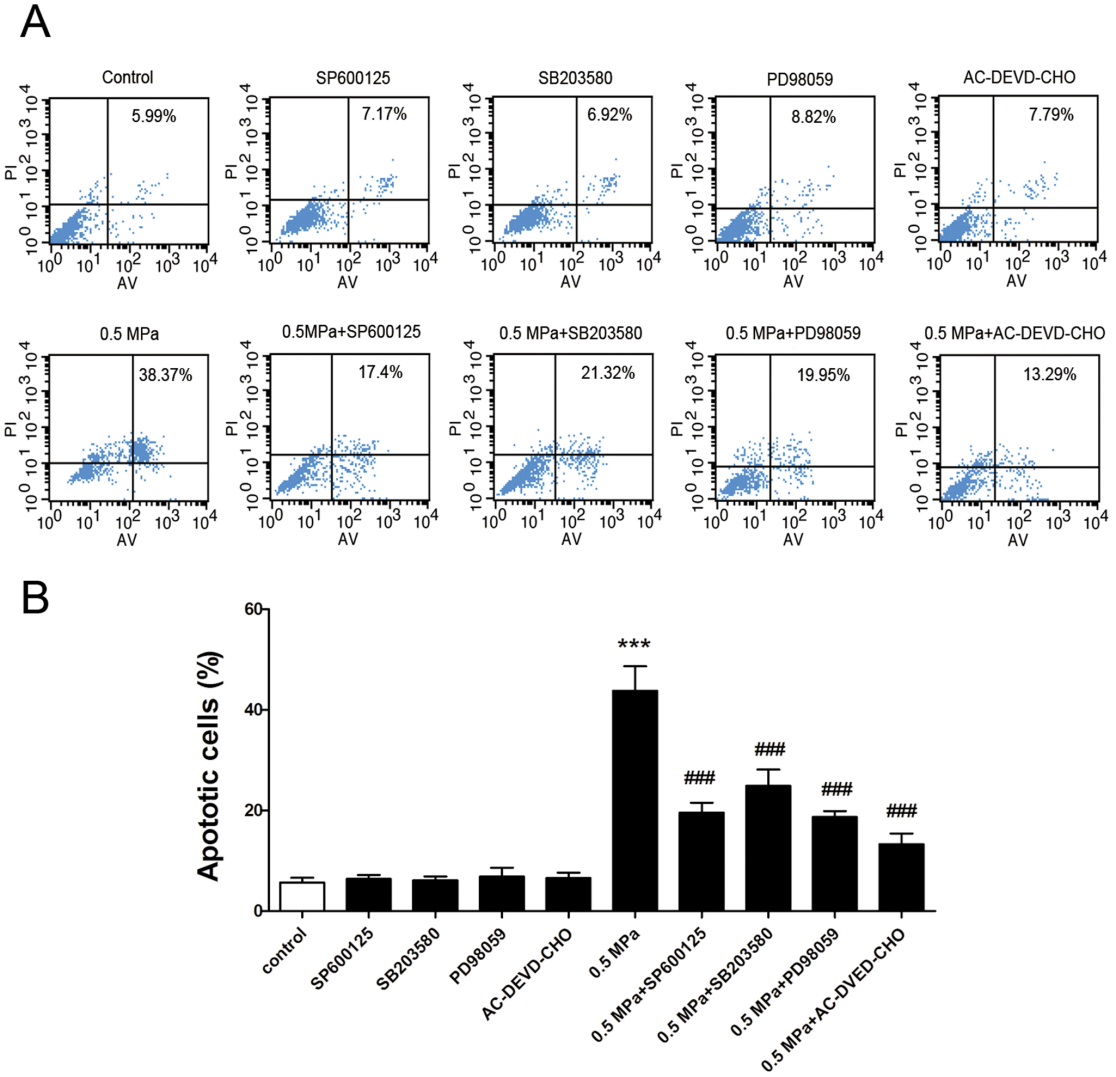
Effect of mechanical stress-induced cell apoptosis in endplate chondrocytes. Chondrocytes were pretreated with or without specific inhibitors for 1 h and then incubated under 0.5 MPa for a further 24 h. (A) Apoptosis was assayed by flow cytometry using annexin-V/PI double staining. (B) The percentage of apoptotic chondrocytes loaded for 24 h. Data are presented as the mean ± SD of three independent experiments. ***p<0.001 versus unloaded cells; ^###^p<0.001 versus loaded cells.

After exposure to 0.5 MPa for 24 h, the frequency of TUNEL-positive cells was 48.77±4.14% in the loaded group, which was significantly higher than that of the control group (4.43±0.85%) (Fig. 2). Apoptosis was also confirmed by flow cytometry using annexin V/PI staining. After exposure to 0.5 MPa for 24 h, the percentage of apoptotic cells in the loaded group increased dramatically compared with the control group (43.79±4.87% vs. 5.66±0.97%) (Fig. 3). Taken together, the results from TUNEL staining and flow cytometry revealed that static compression induces apoptosis in endplate chondrocytes.

### Static Compression Induces Apoptosis via the Mitochondrial Apoptotic Pathway and Activation of Caspases

**Figure 4:**
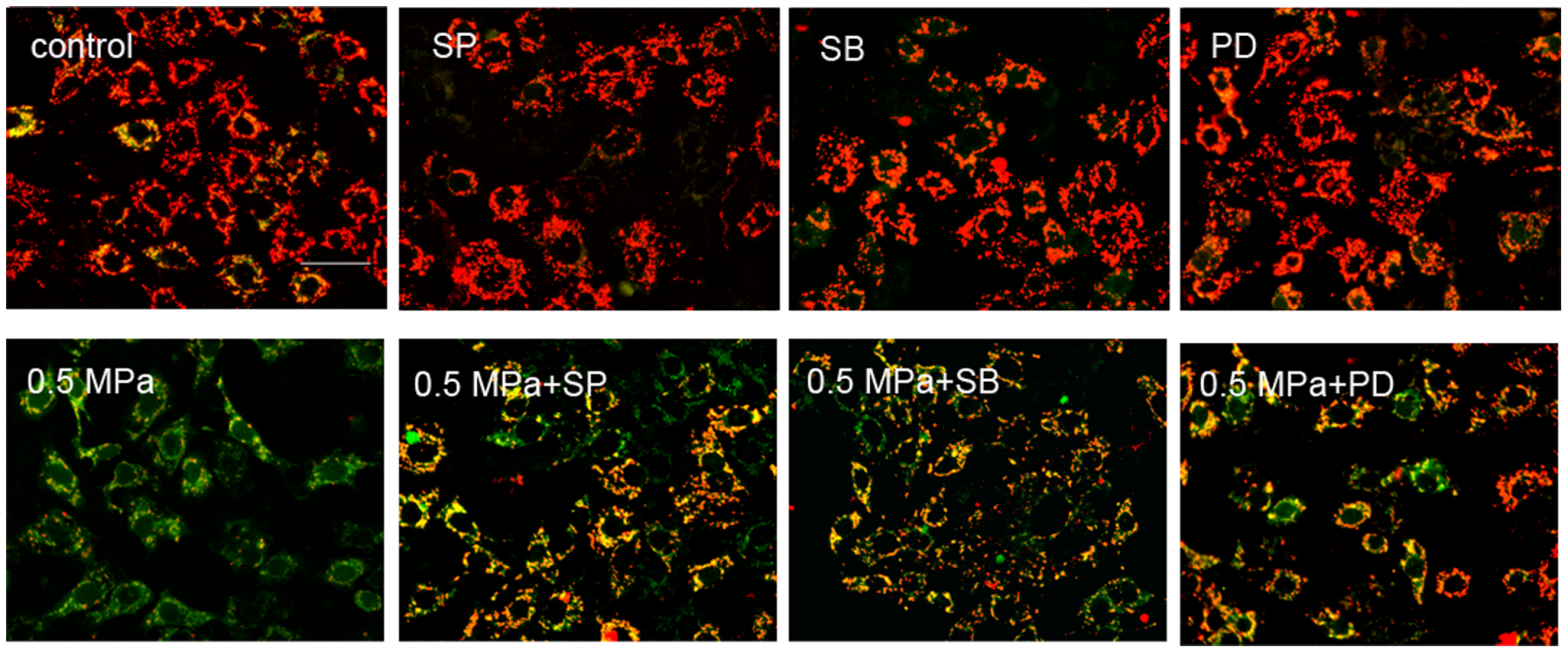
Static compression-induced mitochondrial dysfunction in endplate chondrocytes. Cells were exposed to 0.5 MPa in the presence or absence of specific inhibitors for 24 h, after which the mitochondrial membrane potential was determined by staining with the mitochondrial dye JC-1. Red fluorescence represents JC-1 aggregates formed in healthy cells with high mitochondrial membrane potential, whereas green fluorescence highlights JC-1 monomers in cells with low ΔΨm. Scale bar = 100 µm.

**Figure 5:**
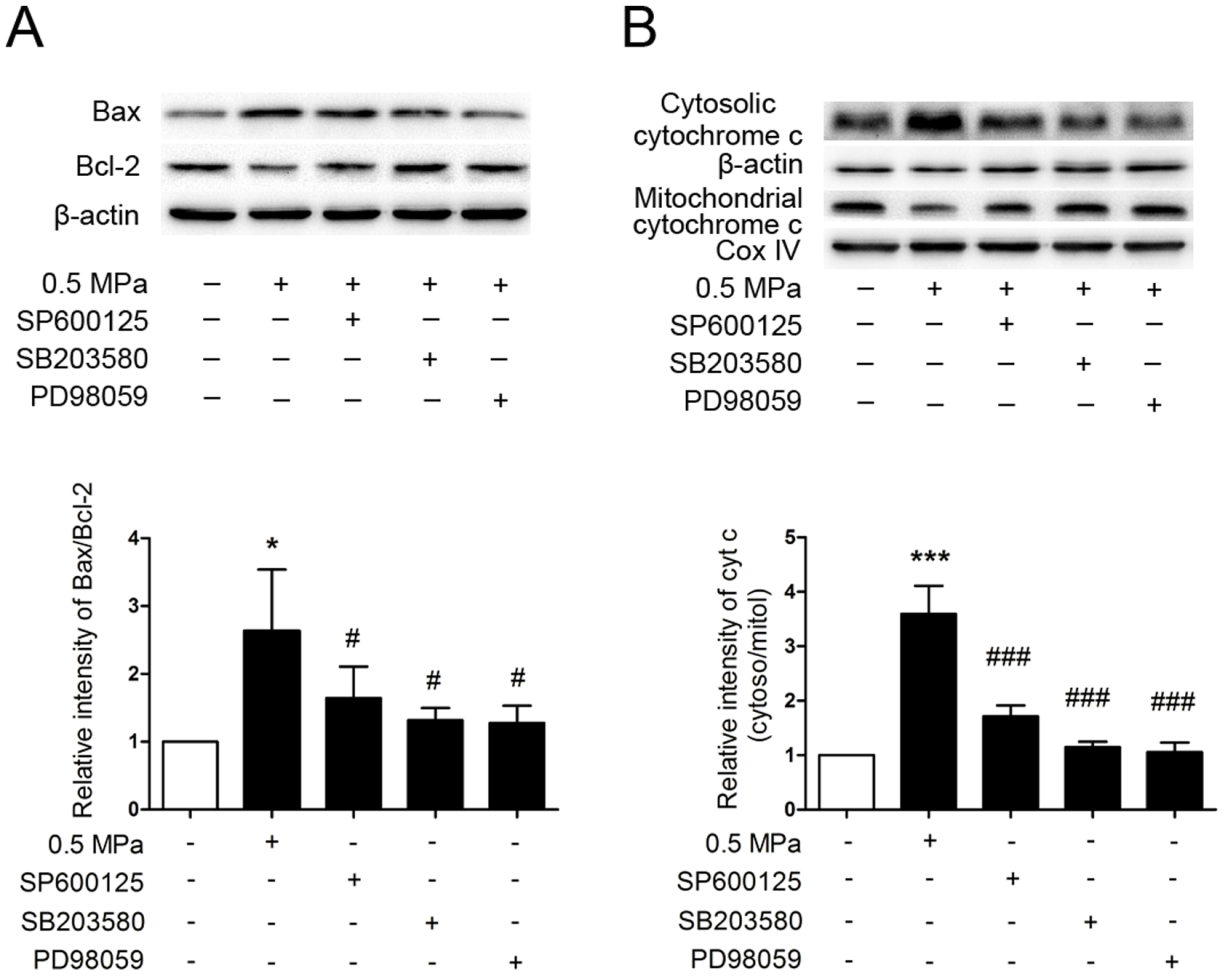
Effect of mechanical stress on the expression of apoptosis-related proteins. Chondrocytes were loaded under 0.5 MPa for 24 h in the presence or absence of specific MAPK inhibitors, and the protein levels of Bax, Bcl-2, and Cytochrome *c* were assessed by western blot analysis. Expression of β-actin was used as an internal control. Data are presented as the mean ± SD of three independent experiments. *p<0.05; **p<0.01 versus unloaded cells, ^#^p<0.05;^ ##^p<0.01; ^###^p<0.001 versus loaded cells.

To investigate the role of mitochondrial dysfunction in static compression-induced chondrocyte apoptosis, we used the lipophilic dye JC-1 to detect the loss of ΔΨm by fluorescence microscopy. As shown in Figure 4, static compression resulted in a decrease of red fluorescence and an increase of green fluorescence in the loaded chondrocytes compared with the control cells, indicative of a loss of ΔΨm. In addition, we evaluated the expression of Bcl-2 family proteins by western blot analysis. As illustrated in Figure 5A, mechanical load treatment resulted in an increased level of Bax protein compared with unloaded cells, whereas the level of Bcl-2 protein was significantly reduced. We next examined the release of Cytochrome *c* from the mitochondria into the cytosol. As shown in Figure 5B, the levels of cytosolic Cytochrome *c* had significantly increased after static compression, indicating that a mitochondrial pathway is involved in mechanical stress-induced chondrocyte apoptosis.

**Figure 6:**
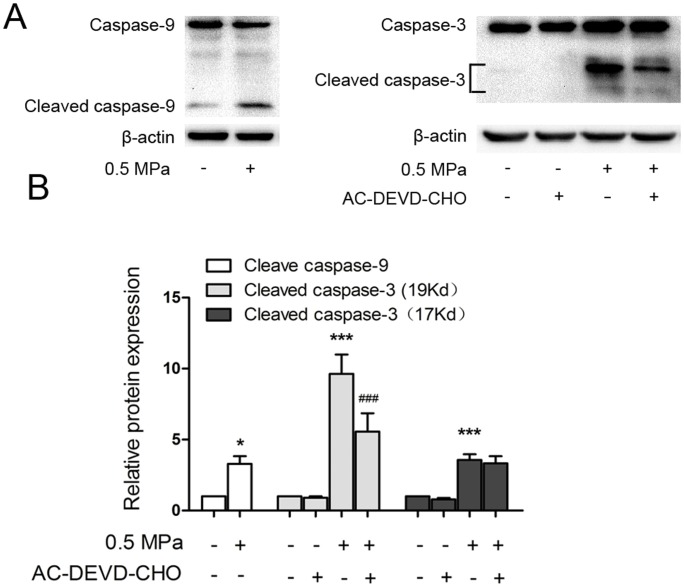
Effect of mechanical stress on the activation of caspases in endplate chondrocytes. (A) Chondrocytes were pre-treated with or without 20 µM Ac-DEVD-CHO for 1 h and then loaded under 0.5 MPa for 24 h. The expression levels of cleaved Caspase-9 and cleaved Caspase-3 were examined by western blotting. (B) Quantitative analysis of cleaved Caspase-9 and cleaved Caspse-3. The results are expressed as the mean ± SD of three separate experiments. *p<0.05; ***p<0.001 versus control cells, ^###^p<0.001 versus loaded cells.

Caspases, a family of cytosolic cysteine proteases, play essential roles in the initiation and execution phases of apoptosis. We used western blot analysis to investigate whether Caspase-9 and Caspase-3 were involved in this process. As shown in Figure 6, the levels of cleaved Caspases 9 and 3 significantly increased after 24 h of treatment. However, pretreatment with 20 µM AC-DEVD-CHO, an inhibitor of Caspase-3 activity, markedly suppressed the expression of cleaved Caspase-3 and significantly decreased the incidence of apoptotic cells induced by mechanical stress. (Fig. 2 and Fig. 3). Taken together, these findings indicate that static load induces chondrocyte apoptosis via a mitochondrial-dependent caspase signaling pathway.

### The Role of the MAP Kinase Pathway in Static Compression-induced Endplate Chondrocyte Apoptosis

**Figure 7:**
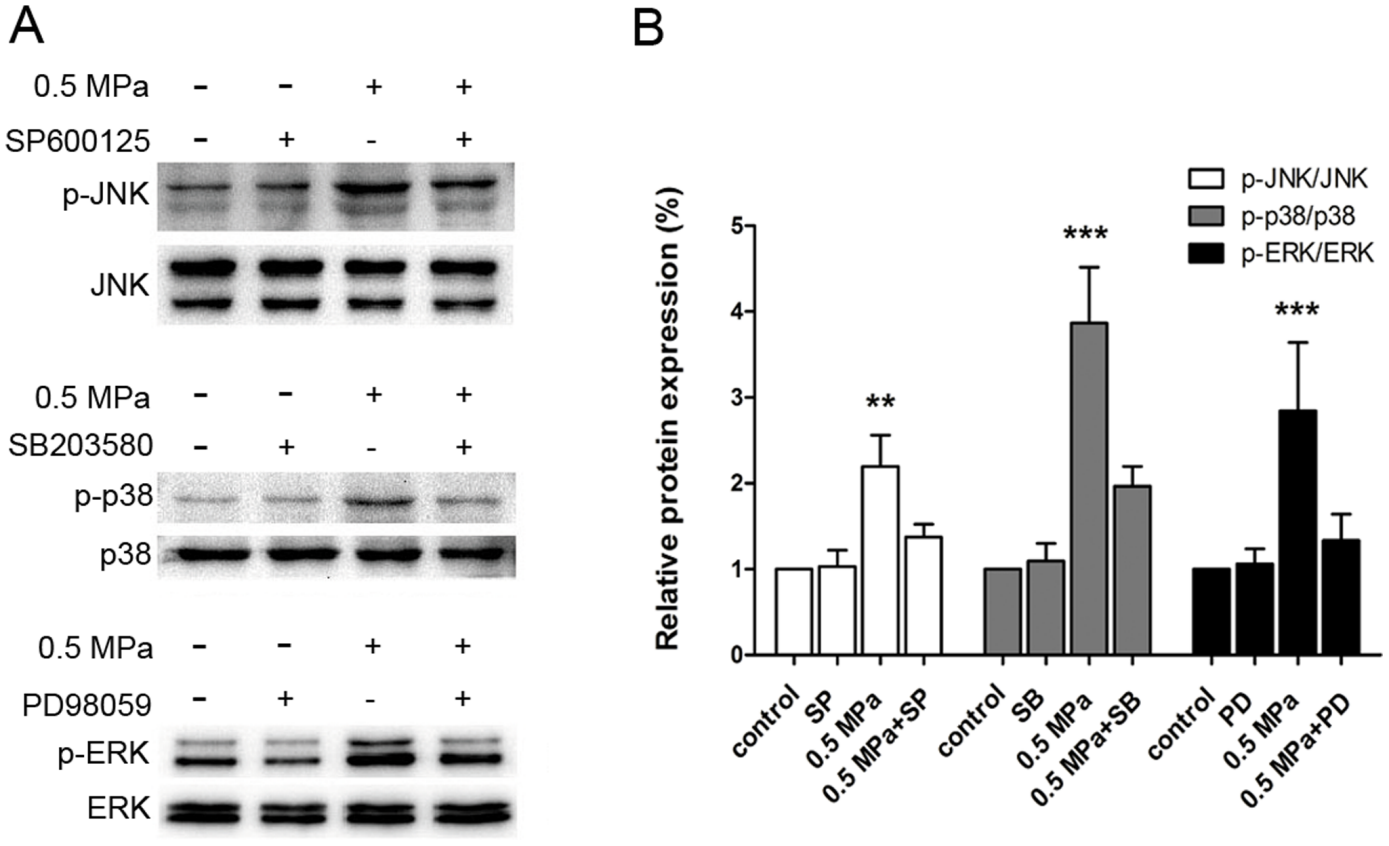
Effect of mechanical stress on phosphorylation of MAPKs. Endplate chondrocytes were loaded (0.5 MPa) for 24 h in the presence or absence of 10 µM SP600125, 10 µM SB203580, and 20 µM PD98059. (A) Western blot analysis of the protein levels of p-JNK, p-p38 MAPK, and p-ERK. (B) Relative intensities are represented as the fold changes of the phosphorylated MAPK protein levels normalized to total MAPK protein levels. Data are presented as the mean ± SD of three independent experiments. **p<0.01; ***p<0.001 versus unloaded cells.

The effects of mechanical stress on the phosphorylation of MAPKs were determined by western blot analysis. As shown in Figure 7, the phosphorylation levels of JNK, p38 MAPK, and ERK1/2 increased dramatically after 24 h of mechanical simulation compared with unloaded cells. Pretreatment of cells with 10 µM SP600125, 10 µM SB203580, and 20 µM PD98059 resulted in significant decreases in the levels of phosphorylated JNK, p38 MAPK, and ERK1/2, respectively compared to loaded cells (Fig. 7).

To further investigate the role of MAPK activation in mechanical load-induced chondrocyte apoptosis, specific inhibitors of MAPK were used to pretreat cells 1 h before mechanical compression. The percentage of apoptotic cells induced by mechanical stress significantly decreased when chondrocytes were pretreated with 10 µM SP600125, 10 µM SB203580, and 20 µM PD98059 (Fig. 2 and Fig. 3). Figure 4 shows that pretreatment with 10 µM SP600125, 10 µM SB203580, and 20 µM PD98059 partially reversed the dissipation of ΔΨm induced by mechanical stress. Western blotting revealed that pretreatment with specific inhibitors of MAPK up-regulated the expression of Bcl-2, down-regulated the expression of Bax, and decreased the release of Cytochrome *c* from mitochondria compared to loaded cells (Fig. 5). These results indicate that static compression induces apoptosis in endplate chondrocytes via a MAPK-dependent mitochondrial apoptotic pathway.

## Discussion

The objective of this study was to investigate the apoptosis signaling pathways induced by mechanical stress. Our study shows that static compression leads to chondrocyte apoptosis via phosphorylation of MAPKs, mitochondrial dysfunction, and activation of caspases.

In previous studies, chondrocyte apoptosis observed in the murine cartilage endplate was induced by static compressive stress, and the number of apoptotic cells increased depending on the magnitude and duration of spinal loading [3], [4]. This is in accordance with our work demonstrating that static mechanical stress induces endplate chondrocyte apoptosis in a dose- and time-dependent manner.

Two main routes leading to apoptosis have been well identified: the death receptor pathway and the mitochondrial pathway [23], [24]. In this study, we focused on the mitochondrial apoptotic pathway induced by mechanical stress. Our results demonstrate that mechanical stress-induced chondrocyte apoptosis occurs through a mitochondrial mechanism as revealed by Cytochrome *c* release from the mitochondria and loss of ΔΨm [25], [26], [27]. The mitochondrial pathway of apoptosis is controlled by the BCL-2 family of proteins, including the anti-apoptotic Bcl-2, Bcl-XL, and Mcl-1 proteins and the pro-apoptotic Bax, Bad, and Bid proteins [28]. Bax exerts pro-apoptotic activity by translocation from the cytosol to the mitochondria, where it induces Cytochrome *c* release, while Bcl-2 exerts anti-apoptotic activity by inhibiting the translocation of Bax to the mitochondria [20], [25]. In this study, mechanical stress stimulation significantly increased Bax protein levels and decreased the expression of anti-apoptotic Bcl-2, which resulted in an increased ratio of Bax/Bcl-2. This imbalance between Bax and Bcl-2 leads to permeabilization of the outer mitochondrial membrane (OMM), release of cytochrome *c* from the mitochondria into the cytosol, and induction of apoptosis [21], [29], [30], [31]. In the present study, we also detected Cytochrome *c* release into the cytosol. Cytochrome *c* in the cytosol can activate Caspase-9, which in turn cleaves and activates the key executioner, Caspase-3, and the sequential activation of Caspase-9 and Caspase-3 leads to apoptosis [22], [28]. In accordance with our observations, we also found that mechanical stress-treated endplate chondrocytes showed increased levels of cleaved Caspase-9 and cleaved Caspase-3, and that the Caspase-3 inhibitor AC-DEVD-CHO significantly decreased the apoptosis level compared with the control group. Taken together, these results suggest that mechanical stress-induced chondrocyte apoptosis is mediated through the mitochondrial pathway.

In the present study, we also investigated whether the MAPK pathway is involved in mechanical stress-induced chondrocyte apoptosis. In general, JNK and p38 MAPK appear to serve roles in cellular responses to stress and cellular damage [14], [32]. Earlier studies have demonstrated that activation of JNK and p38 MAPK can lead to an increase in the mitochondrial translocation of Bax, down-regulation of Bcl-2, and release of Cytochrome *c* into the cytosol [17], [33], [34], [35], [36], [37]. In this study, the mechanical stress-induced chondrocyte apoptosis was accompanied by phosphorylation of ERK1/2, p38 MAPK, and JNK, suggesting that p38 MAPK and JNK activation is mechano-dependent. It is interesting that ERK1/2 was also activated by static compression, because activation of ERK1/2 is usually linked to cellular differentiation and proliferation [38]. Controversy exists regarding the effects of the ERK1/2 signaling pathway. For example, transient ERK1/2 activation is considered to lead to cell proliferation and survival [39], [40]. However, sustained phosphorylation of ERK1/2 is involved in cell apoptosis induced by various stimuli [41], [42], [43], [44]. These results support the hypothesis that sustained phosphorylation of ERK1/2 is involved in cell apoptosis. In addition, specific inhibitors of MAPK were applied to gain further insights into the mechanisms of the MAPK signaling pathway in mechanical stress-induced chondrocyte apoptosis. Pretreatment with specific inhibitors of MAPK effectively blocked the mechanical stress-induced phosphorylation of ERK1/2, p38 MAPK, and JNK. Moreover, mechanical stress-induced mitochondrial dysfunction, Bcl-2 down-regulation, Bax up-regulation, Cytochrome *c* release, and chondrocyte apoptosis were also reversed by pretreatment with specific inhibitors of MAPK. These results indicate that the three MAPK pathways are involved in the endplate chondrocyte apoptosis induced by mechanical stress. However, these results contrast with those reported by Ariga et al., which show that the number of apoptotic cells induced by mechanical stress increased upon treatment with MAPK and p38 inhibitors [4]. These apparent discrepancies may be due to differences in culture conditions, organ-to-monolayer cell culture, or methodology. Further research is needed to understand these discrepancies.

In summary, the results of this study indicate that mechanical stress can induce cell apoptosis in rat endplate chondrocytes, as conformed by annexin V/PI double staining, TUNEL staining, and the activation of Caspase-3. Chondrocyte apoptosis is induced by mechanical stress through the mitochondrial and caspase pathways, as demonstrated by the loss of ΔΨm, the alteration in the ratio of Bax/Bcl-2 protein, Cytochrome *c* release, and the activation of Caspase-9 and Caspase-3. In addition, phosphorylation of JNK, p38, and ERK1/2 was observed after static load treatment. These findings suggest that chondrocyte apoptosis is induced by mechanical stress through the MAPK-mediated mitochondrial pathway.

## Materials and Methods

### Reagents

Phospho-specific antibodies, including phospho-SAPK/JNK (Thr183/Tyr185), phospho-p44/42 MAPK (Erk1/2) (Thr202/Tyr204), and phospho-p38 MAPK (Thr180/Tyr182); antibodies against SAPK/JNK, p44/42 MAPK (Erk1/2), p38 MAPK, Bax, Bcl-2, Caspase-9 and Caspase-3, β-Actin (13E5), and Cytochrome *c*; the horseradish peroxidase-conjugated anti-rabbit secondary antibody, PD98059 (ERK1/2 inhibitor), and the enhanced chemiluminescence (ECL) detection substrate were purchased from Cell Signaling Technology, Danvers, MA, USA. SB203580 (p38 MAPK inhibitor), Ac-DEVD-CHO (Caspase-3 inhibitor), and SP600125 (JNK inhibitor) were purchased from Sigma (St. Louis, MO, U.S.A.). Unless otherwise stated, all chemicals were purchased from Sigma.

### Cell Culture and Mechanical Compression

**Figure 8:**
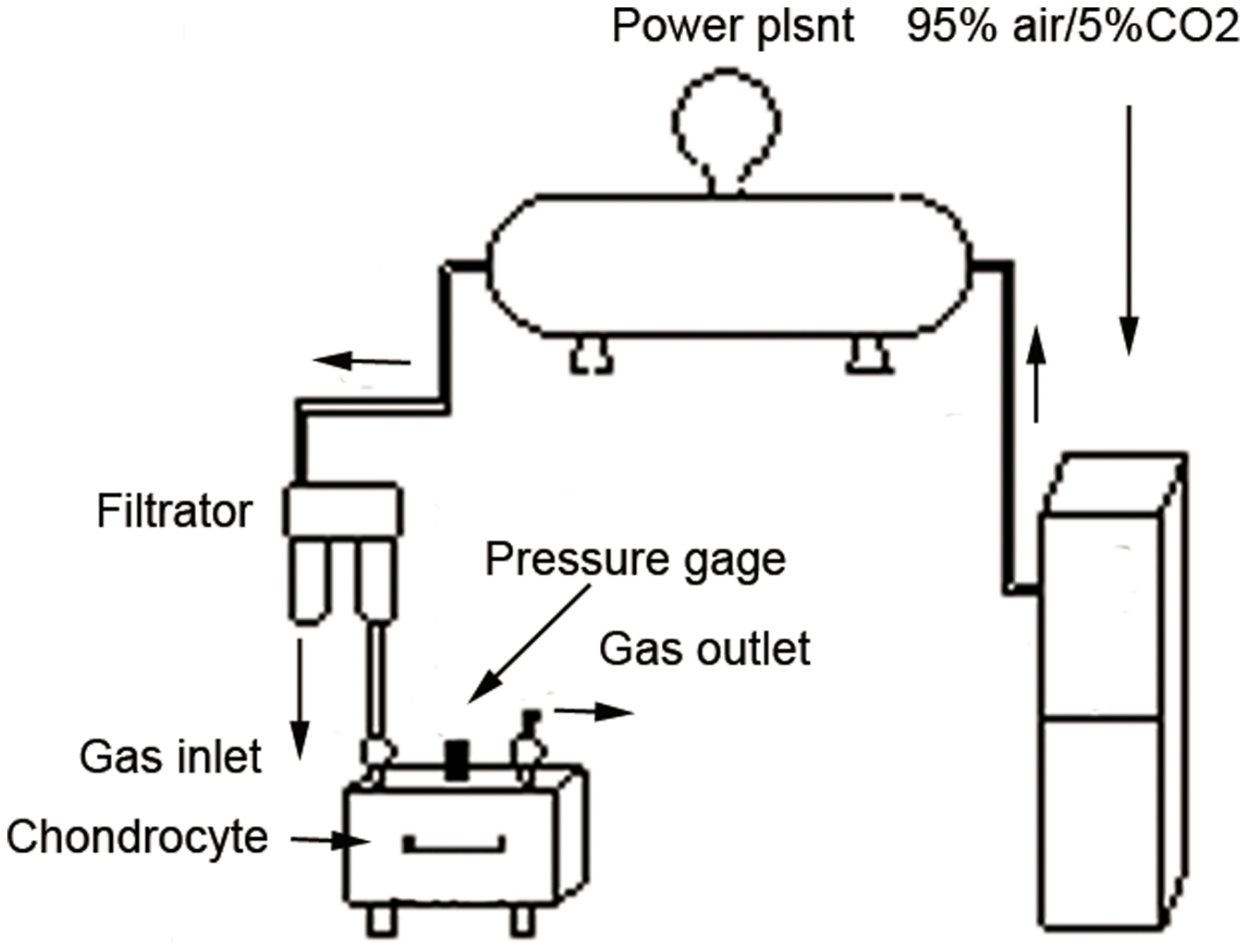
Apparatus used for the static mechanical stress.

Chondrocytes were isolated from the cartilaginous endplates of the cervical vertebrae of Sprague-Dawley rats (100–150 g) under a protocol approved by the animal research committee of Nanjing Medical University. Cartilage tissue was minced and washed in cold phosphate-buffered saline (PBS) and digested with 0.25% trypsin for 30 minutes at 37°C. The slices were then incubated with Dulbecco’s modified Eagle’s medium (DMEM) containing 0.2% collagenase II (Sigma) at 37°C overnight with gentle rocking. Released cells were incubated in a humidified atmosphere incubator containing 95% air and 5% CO_2_ at 37°C in DMEM containing 10% fetal bovine serum and 1% penicillin-streptomycin. All experiments were performed using first-passage cells. Various static mechanical loads were applied to the first-passage chondrocytes in monolayer cultures in a sealed pressure chamber (Fig. 8). The chondrocytes were incubated at 37°C in a humidified atmosphere of 95% air and 5% CO_2_.

### Determination of Cell Viability

Chondrocytes were seeded in 96-well plates at a density of 5×10^3^ cells per well for 24 h, after which cells were starved in serum-free medium for 23 h and then incubated with or without various inhibitors for 1 h before exposure to various static loads for the indicated time intervals. Cell viability was assessed using the Cell-Counting Kit-8 (CCK-8) (Dojindo, Kumamoto, Japan) according to the manufacturer’s instructions. Briefly, after treatment with the static loads, 10 µl CCK-8 was added to each well and incubated at 37°C for 2 h. The optical density was read at a wavelength of 450 nm with a microplate reader (BioTek, USA). Cell viability was calculated using the following formula: optical density of treated group/control group×100%.

### TUNEL Staining

After treatment, apoptosis in endplate chondrocytes was assayed with the In Situ Cell Death Detection Kit (Roche) following the manufacturer’s instructions. Briefly, cells were fixed with 4% paraformaldehyde in PBS for 1 h at room temperature and then permeabilized with 0.1% Triton X-100 in 0.1% sodium citrate for 2 minutes on ice. The cells were then incubated with the reaction mixture for 1 h at 37°C. After rinsing with PBS, cells were stained with DAPI (4′,6-diamidino-2-phenylindole) for 5 minutes and analyzed under a fluorescence microscope.

### Flow Cytometric Analysis

Analysis of the apoptotic cells was also performed after annexin V and propidium iodide (PI) staining of the cells by flow cytometry according to the manufacturer’s protocol (BD PharMingen, San Diego, CA, USA). After treatment, 1×10^6^ cells were harvested and washed three times with PBS, then resuspended in binding buffer followed by Annexin-V/PI labeling at room temperature for 15 min in the dark. The samples were analyzed using a FACScan flow cytometer (BD Biosciences, USA).

### Mitochondrial Membrane Potential (ΔΨm) Assay

The ΔΨm was visualized by fluorescence microscopy using the 5,5′,6,6′-tetrachloro-1,1′,3,3′-tetraethylbenzimidazolcarbocyanine iodide (JC-1; BD) detection kit according to the manufacturer’s instructions. JC-1 accumulates in mitochondria with normal ΔΨm, leading to the formation of JC-1 aggregates and emission of red fluorescence. However, JC-1 leaks out of the mitochondria into the cytoplasm as a monomer when the ΔΨm is depolarized, resulting in a decrease of red fluorescence and an increase of green fluorescence. Endplate chondrocytes were seeded at a density of 1×10^4^ cells on coverslips and treated with a 0.5 MPa static load for 24 h. After treatment, cells were washed in PBS and incubated with JC-1 working solution at 37°C in 5% CO_2_ for 15 min. Stained cells were visualized under a fluorescence microscope (Nikon, Tokyo, Japan).

### Mitochondrial and Cytosolic Fractionation

The mitochondria isolation kit for cultured cells (Pierce) was used to isolate mitochondrial and cytosolic fractions from endplate chondrocytes according to the manufacturer’s protocol. Briefly, 2×10^7^ cells were incubated with 800 µL of mitochondria isolation reagent A and then homogenized on ice in a Dounce homogenizer. Unlysed cells and large debris were pelleted by centrifugation at 700×*g* for 10 minutes at 4°C. The supernatant was further centrifuged at 12,000×*g* for 15 minutes at 4°C. The supernatant (cytosolic fraction) was collected, and the pellet (mitochondrial fraction) was lysed in 2% CHAPS in Tris-buffered saline (TBS; 25 mM Tris, 0.15 M NaCl; pH 7.2).

### Western Blot Analysis

After treatment, cells were washed and lysed in a lysis buffer [20 mM Tris, 150 mM NaCl, 1% NP-40, 1 mM ethylene glycol tetra acetic acid (EGTA), 1 mM PMSF, and 1 mM sodium orthovanadate]. Protein concentrations were quantified using the bicinchoninic acid (BCA) method with the protein assay kit (Thermo Scientific, Rockford, IL). Protein aliquots (30 µg) were separated on 12% SDS-PAGE gels and transferred to polyvinylidene fluoride (PVDF) membranes. After blocking in 5% bovine serum albumin (BSA) in TBS at pH 7.6 with 0.1% Tween-20 (TBST) at room temperature for 1 h, membranes were then incubated with primary antibodies overnight at 4°C. After washing with TBST, blots were then incubated with horseradish peroxidase-linked anti-rabbit secondary antibodies at room temperature for 1 h. Blots were developed using ECL reagents (Cell Signaling Technology) and were then exposed using the digital imaging system(Molecular Imager® ChemiDoc™ XRS+ System), which offers sensitive chemiluminescent detection (Bio-Rad Laboratories Inc., Hercules, CA, USA). The intensity of each band was analyzed using Image Lab 2.0 Software (Bio-Rad Laboratories Inc.,). Intensity values of phosphorylated ERK1/2, p38 MAPK, and JNK were divided by the intensity values of the total protein bands. The intensity of mitochondrial Cytochrome *c* was normalized to that of Cox IV. Unless otherwise stated, β-actin was used as an internal control.

### Statistical Analysis

All results are presented as the mean ± standard deviation (SD). Statistical analysis was performed using one-way analysis of variance (ANOVA) with the Student-Newman-Keuls post hoc test for multiple group comparisons. P<0.05 was considered statistically significant.
